# Design, Synthesis and Evaluation of Branched RRWQWR-Based Peptides as Antibacterial Agents Against Clinically Relevant Gram-Positive and Gram-Negative Pathogens

**DOI:** 10.3389/fmicb.2018.00329

**Published:** 2018-03-02

**Authors:** Sandra C. Vega, Diana A. Martínez, María del S. Chalá, Hernán A. Vargas, Jaiver E. Rosas

**Affiliations:** ^1^Department of Pharmacy, Faculty of Science, Universidad Nacional de Colombia, Bogotá, Colombia; ^2^Laboratory of Public Health, Secretaria Distrital de Salud, Bogotá, Colombia

**Keywords:** antibacterial activity, antimicrobial peptide, cationic peptide, lactoferrin, lactoferricin, multidrug resistance

## Abstract

Multidrug resistance of pathogenic bacteria has become a public health crisis that requires the urgent design of new antibacterial drugs such as antimicrobial peptides (AMPs). Seeking to obtain new, lactoferricin B (LfcinB)-based synthetic peptides as viable early-stage candidates for future development as AMPs against clinically relevant bacteria, we designed, synthesized and screened three new cationic peptides derived from bovine LfcinB. These peptides contain at least one RRWQWR motif and differ by the copy number (monomeric, dimeric or tetrameric) and structure (linear or branched) of this motif. They comprise a linear palindromic peptide (RWQWRWQWR), a dimeric peptide (RRWQWR)_2_KAhx and a tetrameric peptide (RRWQWR)_4_K_2_Ahx_2_C_2_. They were screened for antibacterial activity against *Enterococcus faecalis* (ATCC 29212 and ATCC 51575 strains)*, Pseudomonas aeruginosa* (ATCC 10145 and ATCC 27853 strains) and clinical isolates of two Gram-positive bacteria (*Enterococcus faecium* and *Staphylococcus aureus*) and two Gram-negative bacteria (*Klebsiella pneumoniae* and *Pseudomonas aeruginosa*). All three peptides exhibited greater activity than did the reference peptide, LfcinB (17–31), which contains a single linear RRWQWR motif. Against the ATCC reference strains, the three new peptides exhibited minimum inhibitory concentration (MIC_50_) values of 3.1–198.0 μM and minimum bactericidal concentration (MBC) values of 25–200 μM, and against the clinical isolates, MIC_50_ values of 1.6–75.0 μM and MBC values of 12.5–100 μM. However, the tetrameric peptide was also found to be strongly hemolytic (49.1% at 100 μM). Scanning Electron Microscopy (SEM) demonstrated that in the dimeric and tetrameric peptides, the RRWQWR motif is exposed to the pathogen surface. Our results may inform the design of new, RRWQWR-based AMPs.

## Introduction

The emergence of multidrug-resistant (MDR) bacterial pathogens is a clinically urgent phenomenon that demands the development of new antibiotics (Draenert et al., 2015; Brunetti et al., 2016; da Cunha et al., 2017). Moreover, the incidence of bacteria in healthcare-associated infections (HAIs) is a constantly evolving public health threat that varies geographically (Prakash, 2014). Pathogens currently implicated in HAIs include bacteria such as *S. aureus, K. pneumoniae, P. aeruginosa, E. coli*, and *E. faecalis*, which have widely become multidrug resistant (MDR) (Percival et al., 2015; Brunetti et al., 2016; da Cunha et al., 2017).

Antimicrobial peptides (AMPs) have garnered interest as potential therapeutic agents for MDR infections (Brunetti et al., 2016), especially as they exhibit broad-spectrum activities against diverse strains of Gram-positive and Gram-negative bacteria, including resistant ones, and against fungi (Chung and Khanum, 2017). The rational design of new AMPs offers hope for enhanced biological activity and cheaper, more-efficient production. Rational design methodologies include *in silico* methodologies. Large-scale, high-quality recombinant production can be done using tobacco mosaic virus and gene-editing techniques such as CRISPR (Clustered Regularly Interspaced Short Palindromic Repeats) recombinant peptide biosynthesis (da Cunha et al., 2017).

Evaluation of AMPs usually involves ascertaining how their bioactivity is influenced by physicochemical properties such as the presence of conserved domains; their length, hydrophobicity or hydrophilicity; their structural form (e.g., linear, branched, or cyclic); and their net charges (Shang et al., 2012; de la Fuente-Nunez et al., 2017; Mishra et al., 2017). Previous work has shown that how structural changes to the RRWQWR motif can influence the antimicrobial activity of the resulting peptides (Tam, 1988). Moreover, use of engineered prodrugs and peptide conjugates can improve the specificity of the therapeutic peptide for its intended target.

AMPs with reported antimicrobial activity include peptides derived from the protein bovine lactoferricin B (LfcinB) (Leon-Calvijo et al., 2015). Interestingly, this activity has been attributed to the RRWQWR motif within LfcinB, which is considered to be the smallest known motif with antibacterial (Richardson et al., 2009; Leon-Calvijo et al., 2015; Huertas et al., 2017) or anticarcinogenic (Solarte et al., 2015) activity.

In the present work, we sought to better understand the contribution of the RRWQWR motif to the antimicrobial activity of LfcinB-derived AMPs, so that we could obtain new, lactoferricin B (LfcinB)-based synthetic peptides as viable early-stage candidates for future development as AMPs against clinically relevant bacteria. To this end, we designed, synthesized and screened a set of cationic LfcinB-based peptides that contain at least one motif RRWQWR and that vary by the copy number and structure of this motif. After preparing these peptides by solid-phase peptide synthesis, we screened them against various bacterial cell lines from ATCC and against clinical bacterial isolates relevant to HAIs. This enabled us to identify two peptides with attractive biological and physicochemical profiles that could ultimately inform a new generation of antibiotics.

## Materials and methods

### Microorganisms

We sought to assess antibiotic-sensitive and antibiotic-resistant strains of representative Gram-positive and Gram-negative bacteria from the American Type Culture Collection (ATCC). Accordingly, we chose *E. faecalis* as the Gram-positive species (lines ATCC 29212 and ATCC 51575 as sensitive and resistant, respectively) and *P. aeruginosa* as the Gram-negative species (lines ATCC 10145 and ATCC 27853 as sensitive and resistant, respectively). All strains were purchased from ATCC.

For the clinical isolates, we used 20 different isolates from the Public Health Reference Laboratory collection of the Secretaría de Salud del Distrito (SdSD; Bogotá, Colombia). The samples were collected from June to December 2016. For each isolate, the patient parameters (age, gender and location) and the culture site were recorded for epidemiologic monitoring (Table 1). All isolates had been previously tested for antibiotic sensitivity at the Public Health Microbiology Laboratory using either the Phoenix™ system (Gram-positive) or the VITEK 2 system (Gram-negative).

**Table 1:** The clinical isolates of HCAI-relevant bacteria used in this study.

**Gram classification**	**Species**	**Isolate**	**Age (years)/sex**	**Clinical service**	**Origin**
Gram-positive	*E. faecium*	550	1 (M)	ICU	Blood
		1,040	39 (F)	Surgical unit	Brain tumor
		1,225	58 (F)	Medical unit	Urine
		1,461	26 (M)	Observation	Skin
		1,462	80 (M)	ICU	Peritoneal liquid
	*S. aureus*	52,013	63 (F)	Medical unit	Body fluids
		43,062	69 (F)	ICU	Trachea
		43,337	22 days (F)	Emergency unit	Eye
		48,575	41 (M)	Hematology	Blood
		48,577	42 (F)	Medical unit	Secretion ulcer
Gram-negative	*K. pneumoniae*	49,644	69 (M)	Medical unit	Blood
		50,181	59 (M)	ICU	Bronchoalveolar lavage
		50,424	32 (F)	ICU	Abdominal wall secretion
		51,048	72 (M)	ICU	Blood
		51,009	47 (M)	Medical unit	Urine
	*P. aeruginosa*	47,661	65 (F)	Medical unit	Catheter
		48,220	81 (M)	Medical unit	Urine
		48,221	76 (M)	Medical unit	Urine
		48,458	94 (M)	ICU	Urine
		48,526	55 (F)	Medical unit	Urine

*From the Public Health Reference Laboratory collection of the Secretaría de Salud del Distrito (SdSD; Bogotá, Colombia). Samples gathered from July to December 2016*.

### Antibacterial peptides

**Figure 1:**
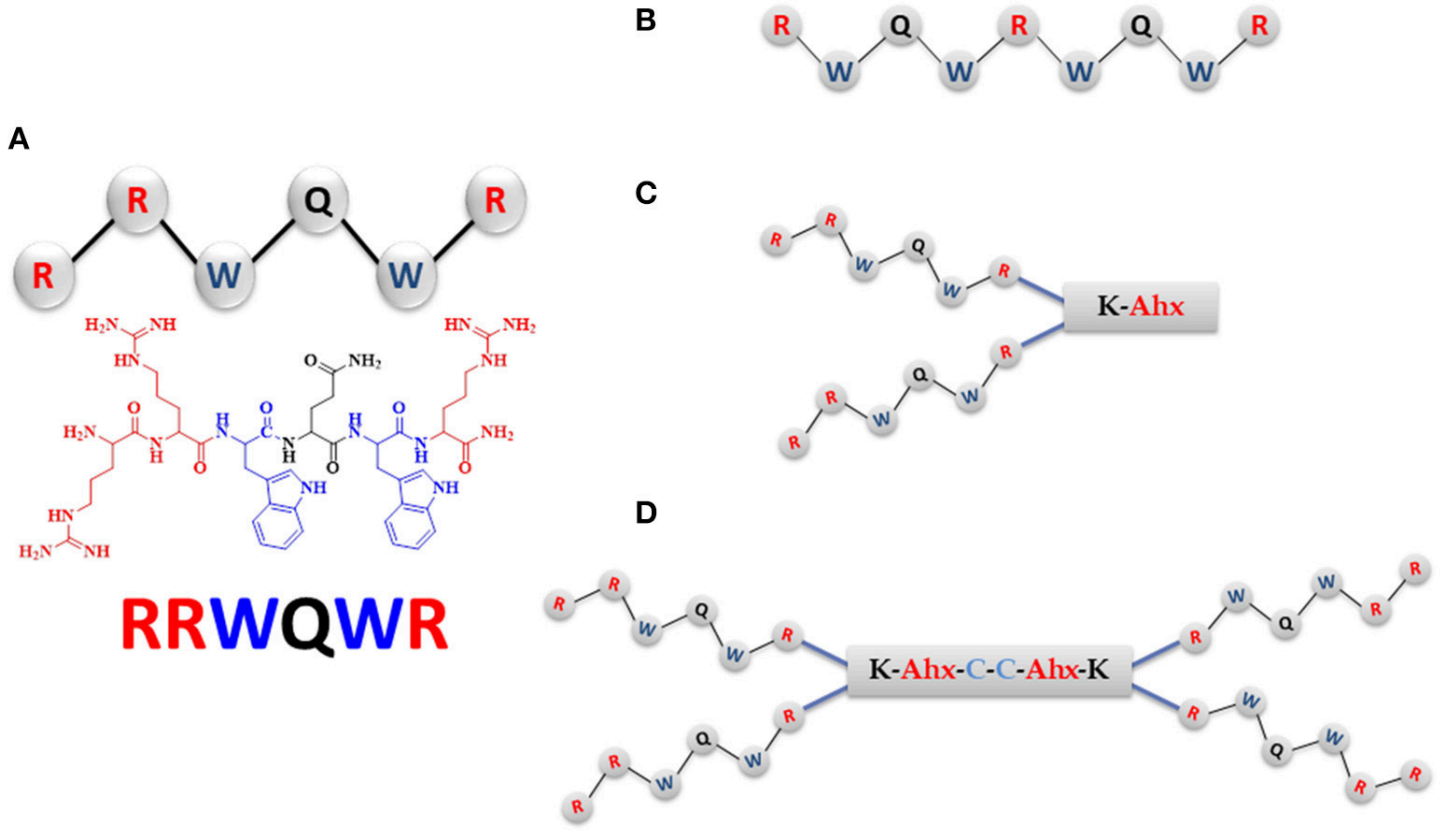
The RRWQWR-based peptides designed, synthesized, and screened for antibacterial activity. In blue: hydrophobic amino acids; in red: cationic amino acids. **(A)** Linear monomer. **(B)** Linear palindromic peptide. **(C)** Branched dimeric peptide, in which the two monomers are linked to a tripeptide comprising Lys, Ahx (in red) and terminating in Cys (in blue). **(D)** Branched tetrameric peptide, comprising two of the peptides shown in **(C)** linked by a cysteine disulfide bridge (in blue).

We designed and synthesized three new cationic peptides based on the RRWQWR motif and prepared two other peptides for comparison (hy): LfcinB (20–25) (RRWQWR) and LfcinB (17–31) (FKCRRWQWRMKKLGA), the latter as reference peptide or antibacterial activity, based on results previously reported by Leon-Calvijo et al. (2015). All peptides were synthesized on solid phase using the Fmoc/tBu methodology, as previously reported (Solarte et al., 2015; Huertas et al., 2017). The sequences described in Table 2 were synthesized by Fmoc/tBu solid-phase peptide synthesis, as previously reported (Shang et al., 2012; Percival et al., 2015; de la Fuente-Nunez et al., 2017; Mishra et al., 2017). The steps are listed below. Firstly, the solid support, Rink-amide resin (0.66 meq/g substitution), was swelled with dimethylformamide (DMF) for 2 h at room temperature with constant stirring. Next, the resin was treated with a 20% solution of 4-methylpiperidine in DMF to remove the Fmoc group, to enable coupling of the first amino acid. For all coupling steps, the desired Fmoc-protected amino acid was first pre-activated with DCC/HOBt (0.20 mmol/0.21 mmol) in DMF, and then added to the deprotected resin. Each coupling reaction was monitored using the ninhydrin test. Once coupling was complete, the terminal Fmoc-group of the newly added amino acid was removed as above. Iterative coupling and deprotection was performed until the desired peptide sequence was obtained. Finally, the side chains were deprotected as follows: firstly, the peptide was cleaved from the solid support using “cleavage” cocktail containing (TFA/water/ Triisopropyl silane (TIS)/EDT (93/2/2.5/2.5% v/v). The reaction was stirred for 6 h (for some sequences up to 12 h) at RT, and then the mixture was filtered and the solution was collected. Next, the peptide was precipitated out with cold ethyl ether, and finally, it was purified by extraction in solid phase. All peptides were characterized by reverse-phase, high-performance liquid chromatography (RP-HPLC) and mass spectrometry. To obtain the dimeric peptide, di-FMOC-protected lysine was used, which enabled simultaneous synthesis of the two peptide chains (one from the α-amino group and the other, from the ε-amino group of this amino acid). The tetrameric peptide was obtained via oxidation of the dimeric peptide, (RRWQWR)_2_-K-Ahx-C, with 10% DMSO % in PBS buffer (pH 7.5), as described by Leon-Calvijo et al. (2015), which led to formation of a disulfide bond between the side chains of the cysteine residues at the carboxyl terminus (Figure 1). All peptides were >90% pure (as determined by RP-HPLC) and had the expected molecular weight (determined by MALDI-TOF MS). The peptides were synthesized by the SAMP research group of the Faculty of Science of the Universidad Nacional de Colombia and stored in lyophilized form.

**Table 2:** Structure and physicochemical properties of the cationic peptides used in this study.

**Alternate name**	**Sequence**															**RP-HPLC**	**MALDI-TOF (M/Z) [M**+**H]**^**+**^		**^b^Net charge**	**Residues**	**Hydro-phobic amino acids (%)**	**^b^GRAVY**
																**^a^t_R_(Min)**	**Theor**.	**Exper**.				
Motif				^20^**R**	**R**	**W**	**Q**	**W**	**R**^25^							4.33	985.55	986.66	+3	6	33.3	−3.133
Lineal/palindromic					**R**	**W**	**Q**	**W**	**R**	W	Q	W	R			5.95	1,485.75	1,488.58	+3	9	44.4	−2.678
Lfc B reference Peptide	^17^F	K	C	**R**	**R**	**W**	**Q**	**W**	**R**	M	K	K	L	G	A^31^	5.25	1,992.09	1,994.71	+6	15	33.3	−1.207
Dimeric				**(R**	**R**	**W**	**Q**	**W**	**R)**_2_	K	Ahx					5.21	2,195.24	2,198.51	+6	15	26.7	–
Tetrameric				**(R**	**R**	**W**	**Q**	**W**	**R)**_4_	K_2_	Ahx_2_	C_2_				19.11	2,298.32^c^	2,302.96^c^	+12	30	26.7	–

a *tR: Retention time of the main product (in minutes)*.

b *Net charge values and Grand Average of Hydropathy (GRAVY) values were calculated using the Antimicrobial Peptide Calculator and Predictor (http://aps.unmc.edu/AP/prediction/prediction_main.php). However, this was not possible for the branched peptides*.

c *Experimental molecular weight that correspond to dimeric molecule before oxidation*.

### Screening for antibacterial activity

We screened all five peptides against the ATCC reference strains and the clinical isolates according to Method M7-A7 of the National Committee for Clinical Laboratory Standards (CLSI, 2007). The MIC_50_ and MBC values were determined using a broth microdilution and growth inhibition method previously reported by Leon-Calvijo et al. (2015), with some modifications (Wiegand et al., 2008). Briefly, the MIC_50_ experiments comprised a liquid-inhibition growth assay in a sterile, untreated, 96-well flat-bottom tissue culture plate. The bacteria were cultured overnight n Mueller Hinton agar; three colonies were transferred to 8 mL of Mueller Hilton broth and incubated at 37°C until the mid-exponential phase of growth. The turbidity of the cultures was measured and adjusted spectrophotometrically to a McFarland standard of 0.5, and then diluted to a final concentration of 5 × 10^7^ colony forming units (CFU) per well. Stock solutions (2,000 μM) of each test peptide were serially diluted to final concentrations (per well) of 200, 100, 50, 25, 12.5, and 6.25 μM. Each concentration was evaluated in duplicate and each assay was performed in triplicate.

Wells containing Mueller Hilton broth with bacterial inoculum only served as bacterial-growth controls. Additional controls included Mueller Hilton broth alone (as blank) and Mueller Hilton broth with ciprofloxacin (2 μg/mL; as positive control). The microplate was incubated for 24 h at 37°C, and growth inhibition was measured by monitoring the optical density at 620 nm (OD_620_). The MIC_50_ was defined as the peptide concentration at which bacterial growth was inhibited by 50%.

To determine the MBC, an aliquot from each well of the MIC_50_ assay was spread onto Mueller Hilton agar. After 18 h at 37°C, the concentration that inhibited bacterial growth was determined. Each of these tests was performed four times. MBC was defined as the lowest concentration of peptide at which the number of bacteria was reduced by 99.9% *in vitro* (European Committee for Antimicrobial Susceptibility Testing, 2000).

### Scanning electron microscopy

We observed bacterial morphology by SEM. The *E. faecalis* and *P. aeruginosa* strains were grown to mid-logarithmic phase, and adjusted spectrophotometrically to a McFarland standard of 0.5 (corresponding to ~1 × 10^8^ CFU/mL). Subsequently, 1 mL of bacterial suspension was distributed into three tubes: one tube was treated with (RRWQWR)_2_KAhx at 3 × the MIC_50_; another tube, with (RRWQWR)_4_K_2_Ahx_2_C_2_ at the same concentration; and the third tube was left untreated, as a control. The samples were incubated aerobically at 37°C for 2 h, and the bacterial suspensions were centrifuged at 1,459 × g for 3 min and then, washed twice with Millonig's Phosphate Buffer (0.10 M, pH 7.4). For SEM, each sample was fixed with 1 mL of 2.5% glutaraldehyde at 4°C for 2 h. The fixed samples were dehydrated in an ethanol gradient (50, 70, 80, 90, and 100%) for 20 min and then, centrifuged at 1,459 × g for 10 min. The bacterial pellet was resuspended in 100% ethyl alcohol and air-dried. Finally, the slides were taped onto stubs, coated with gold using a Quorum Q150R sputter coater, and observed with an FEI Quanta 200-r microscope.

### Hemolytic activity

Human erythrocytes collected from the blood samples of healthy humans were harvested by centrifugation for 7 min at 162 × g and washed three times in phosphate-buffered saline (PBS). The erythrocytes (2% hematocrit in PBS) were incubated with peptide molecules at several concentrations (6.25, 12.5, 25, 50, and 100 μM) for 2 h at 37°C. PBS was used as negative control for hemolysis, and sterile distilled water was used as positive control (100% hemolysis). The plate was subsequently centrifuged at 1,459 × g for 10 min at 4°C. Aliquots of the supernatant from each well (75 μL) were carefully transferred to a new sterile 96-well plate, and hemolytic activity was evaluated by measuring the OD_492_ using an Asys Expert Plus Microplate reader. The experiments were performed in duplicate, and hemolytic activity was calculated for each peptide.

### Therapeutic index

We determined the therapeutic index of each peptide, which we defined as the ratio of Maximum Hemolytic Activity (H_max_) to MIC_50_ (H_max_/MIC_50_).

### Statistical analysis

We analyzed all the data using SPSS 11.0 software. The results are presented here as the mean ± standard deviation. MIC_50_ values were determined by interpolation on a four-parametric curve of pharmacology functions.

## Results

### Antibacterial peptides

The crude products were characterized using RP-HPLC and then purified. The chromatogram of each purified product exhibited a primary peak corresponding to the desired peptide (purity: > 90%). The molecular weight of each peptide was confirmed by MALDI-TOF-MS (Table 2). Stock solutions of each peptide were prepared in water (2,000 μM), sterilized by 0.22 μm filtration, and stored at −20°C until used in the subsequent experiments.

### Antibacterial assay: ATCC strains

**Figure 2:**
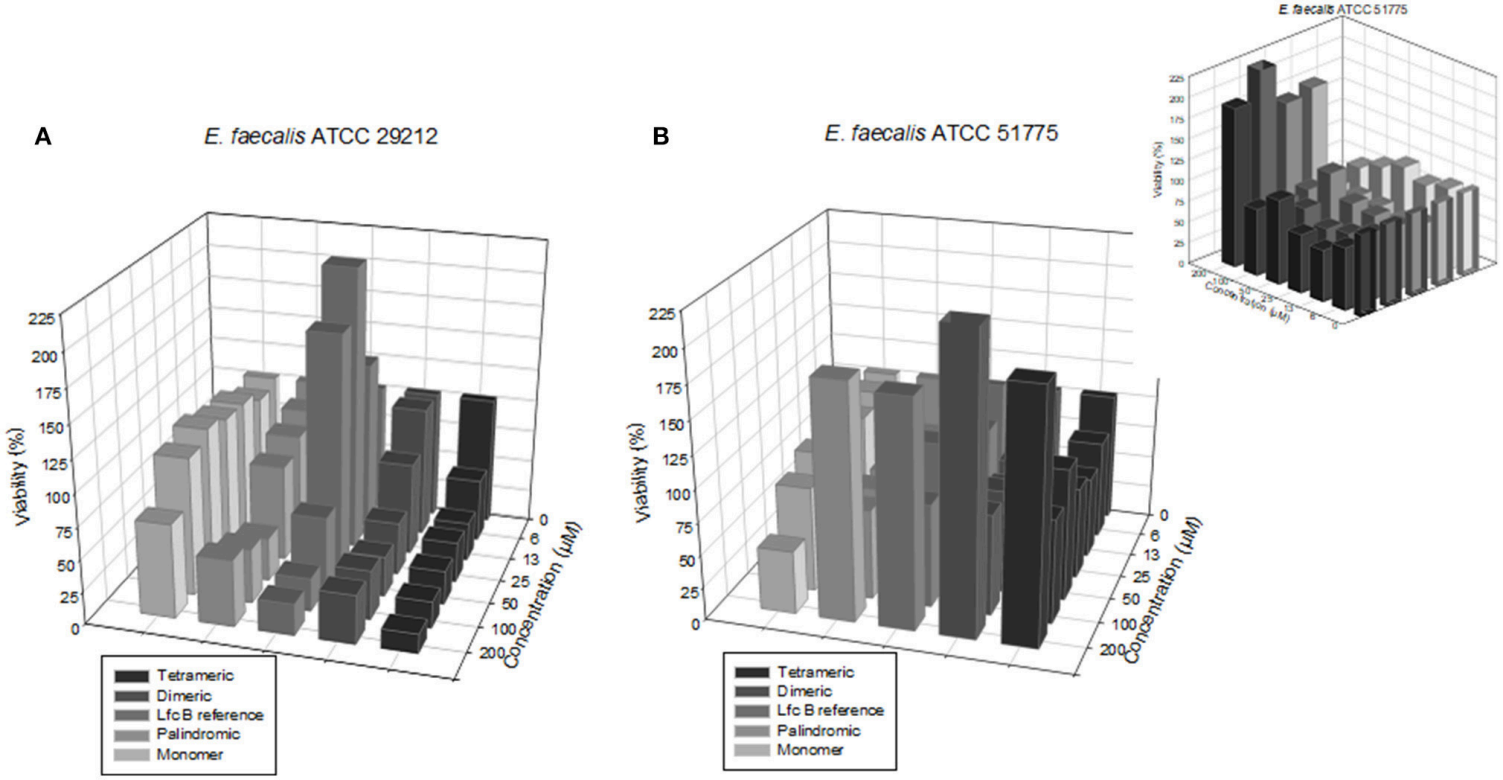
Dose-response plots of the antibacterial activity of the test peptides against the two *E. faecalis* strains. **(A)** Sensitive strain. **(B)** Resistant strain. (**B**: inset) Plot seen from a different perspective, revealing that at higher concentrations, all of the peptides except the monomer induced strong bacterial proliferation.

The screening results for each peptide against the sensitive and resistant strains of *E. faecalis* are shown in Figure 2, which shows that the activities varied by peptide and by strain. Activity was assessed in terms of bacterial viability, whereby the control (untreated) samples showed a viability of 100%. As shown in Figure 2A, against the sensitive strain, the highest activity (lowest viability value) observed for each peptide was: for the RRWQWR monomer, 72.8% at 200 μM; for the palindromic peptide, 33.3% at 50 μM; for the dimeric peptide, 40.9% at 25 μM; for the tetrameric peptide, 48.9% at 6.2 μM; and for the reference peptide (LfcinB), 25.6% at 100 μM. Overall, the RRWQWR monomer appeared to be the weakest antibacterial agent. However, and rather curiously, for the samples treated with LfcinB at 6.25, 13.0, and 25.0 μM, the bacterial viability was actually higher than for the untreated sample. As shown in Figure 2B, against the resistant strain of *E. faecalis*, the highest activity (lowest viability value) observed for each peptide was: for the RRWQWR monomer, 48.4% at 200 μM; for the palindromic peptide, 61% at 12.5 μM; for the dimeric peptide, 65.3% at 25.0 μM; for the tetrameric peptide, 62.4% at 12.5 μM; and for the reference peptide (LfcinB), 8.3% at 200 μM. Overall, the most active peptide appeared to be the tetramer. Studying the dose-response plot of 1B from another perspective (Figure 1B, inset), reveals two important findings: firstly, that these peptides are generally inactive against the resistant strain of *E. faecalis*; and secondly, that at the highest concentration, all of them except for the monomer induced strong bacterial proliferation.

The experiments on *E. faecalis*, Figure 2 revealed three major findings: firstly, that the most active peptides were the tetrameric peptide and the dimeric peptide; secondly, that at most concentrations, the monomer was inactive against both strains; and lastly, that at some concentrations, some of these peptides actually induced proliferation of either strain. Overall, the palindromic and Lfc B peptides exhibited significant antimicrobial activity with the higher concentration evaluated in this study (200 μM). The dimeric peptide and the tetrameric peptide exhibited the strongest antimicrobial activity on each strain at the lowest concentrations (50 and 25 μM, respectively).

**Figure 3:**
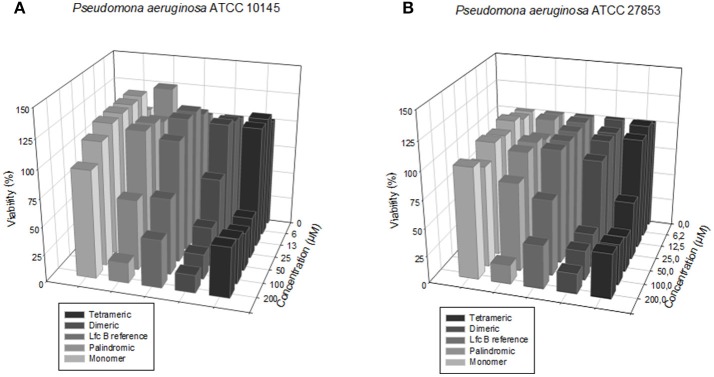
Dose-response plots of the antibacterial activity of the test peptides against the two *P. aeruginosa* strains. **(A)** Sensitive strain. **(B)** Resistant strain.

The screening results for each peptide against the sensitive and resistant strains of *P. aeruginosa* are shown in Figure 3.

We calculated the MIC_50_ values for each peptide against the sensitive and resistant strains of *E. faecalis* and of *P. aeruginosa*, using a broth microdilution assay. The values are shown in Table 3. In terms of activity against all four bacterial strains, the peptides ranked, from most active to least active, as follows: tetrameric > dimeric > palindromic > reference > monomer.

**Table 3:** Antibacterial activity of the RRWQWR-based peptides against the ATCC strains of HCAI-relevant bacteria.

**Bacterium**		***Enterococcus faecalis***				***Pseudomonas aeruginosa***			
**Strain ATCC#**		**Sensitive 29212**		**Resistant 51575**		**Sensitive 10145**		**Resistant 27853**	
**Peptide**	**Alternate name**	**MIC_50_ (μM)**	**MBC (μM)**	**MIC_50_ (μM)**	**MBC (μM)**	**MIC_50_ (μM)**	**MBC (μM)**	**MIC_50_ (μM)**	**MBC (μM)**
LfcinB (20–25)	Monomer	>200.0	>200.0	198.0	>200.0	>200.0	>200.0	>200.0	>200.0
PLS	Palindromic	25.6	100.0	>200.0	>200.0	99.7	>200.0	107.2	>200.0
LfcinB (17–31)	Reference	34.3	>200.0	>200.0	>200.0	111.7	>200.0	99.6	>200.0
LfcinB (20–25)_2_	Dimeric	13.1	100.0	>200.0	>200.0	29.1	100.0	34.8	200.0
LfcinB (20–25)_4_	Tetrameric	3.1	25.0	>200.0	>200.0	18.1	50.0	21.7	50.0

Importantly, the RRWQWR monomer was generally inactive against all *E. faecalis* and *P. aeruginosa* strains (MIC_50_ > 200 μM); moreover, it exhibited a MIC_50_ of 198 μM against the resistant strain of *E. faecalis*. Importantly, against the resistant strain of *E. faecalis*, none of the other peptides exhibited any activity (MIC_50_ > 200 μM). The reference peptide (LfcinB) exhibited a similar profile to that of the monomer, except against the sensitive strain of *E. faecalis*, against which it was moderately active (MIC_50_ < 50 μM). Intriguingly, the palindromic, dimeric and tetrameric peptides were each more active against the Gram-positive bacteria than against the Gram-negative bacteria. These experiments demonstrated that in the range of concentrations tested, all of the peptides showed at least some activity against at least one of the bacterial lines, with the palindromic, dimeric and tetrameric peptides generally the most active.

We calculated the MBC values for each peptide, which showed the activity against the sensitive *E. faecalis* strain (or both strains) relative to the corresponding value(s) for the tetrameric peptide (MBC_tet_), as it was the most active one (e.g., MBC_tet_ against the sensitive *E. faecalis* strain: 25.0 μM). Thus, the activity ranking for the three active peptides is: tetramer (MBC_tet_) > dimer (4 × MBC_tet_) = palindromic (4 × MBC_tet_). The MBC of this peptide against the sensitive *P. aeruginosa* strain was 25.0 μM. Therefore, the activity ranking for the two active peptides is: tetramer (MBC_tet_) > dimer (4 × MBC_tet_). Finally, the MBC of the tetrameric peptide against the resistant *P. aeruginosa* strain and 4 × MBC for the resistant strain, which gives an activity ranking of: tetramer (MBC_tet_) > dimer (4 × MBC_tet_).

### Scanning electron microscopy (SEM)

We used SEM to study the morphology of bacterial cells before and after treatment with either branched peptide (dimeric and tetrameric). To this end, each strain of *E. faecalis* and *P. aeruginosa* was first studied by SEM; then, independently treated in the exponential phase with either peptide at 3 × the corresponding MIC_50_ value for 2 h (except for the resistant *E. faecalis* strain, for which a peptide concentration of 200 μM was used); and finally, studied by SEM again.

### E. faecalis

Before treatment, the antibiotic-sensitive *E. faecalis* cells were spherical or ovoid, had a smooth surface and exhibited a primarily diplococcic structure; the untreated antibiotic-resistant *E. faecalis* cells had a similar appearance but exhibited little surface mucus (Figure S1). After treatment with the dimeric peptide, the sensitive *E. faecalis* cells exhibited a random organization with morphological alterations (e.g., pitted and wrinkled surface) and alterations to cell-membrane surface morphology and agglutination, which might have caused leakage of cellular contents. In contrast, treatment of sensitive *E. faecalis* cells with the tetrameric peptide induced population decline, cell-size heterogeneity and cell-surface alterations in the form of protrusions. Treatment of the resistant *E. faecalis* cells with either of these peptides induced alterations in the surface mucus levels and, in some cases, morphologic alterations (e.g., amorphous cells or surface changes, in the case of the tetrameric peptide); however, there were no changes in population.

### P. aeruginosa

Before treatment, the untreated antibiotic-sensitive *P. aeruginosa* cells were uniformly rod-shaped and exhibited intact cell membranes (Figure S1). However, treatment with the dimeric peptide induced a clear reduction in population and caused morphological alterations (e.g., wrinkling and surface shrinkage). Treatment of this strain with the tetrameric peptide led to a very heterogeneous population and to alterations in the cell surface, namely in the form of protrusions, pores and disrupted membranes. Moreover, the tetrameric peptide induced a total transformation of cell morphology, from rod-shaped to spherical, and led to aggregation of diversely sized spheres. Before treatment, the antibiotic-resistant *P. aeruginosa* cells resembled those of the sensitive strain, but were slightly longer and exhibited surface mucus. Treatment with the dimeric peptide caused a marked drop in population and severe morphological alterations (e.g., cell elongation and cell-membrane porosity). Treatment with the tetrameric peptide was even more dramatic, leading to disintegrated and irregularly-shaped mucoid cells that exhibited surface changes and to heterogeneous aggregates. Importantly, in both strains of *P. aeruginosa*, both treatments appeared to induce leakage of cellular contents that may have contributed to the observed aggregation.

### Hemolytic activity

To evaluate the effects of all five test peptides on normal human erythrocytes, we independently treated erythrocytes with each of the five test peptides, using the standard microtiter dilution method (Table 4). For all peptides, the H_50_ was > 100 μM. However, the H_max_ values demonstrated a clear ranking of hemolytic activity for the peptides, from strongest to weakest: tetrameric > palindromic > monomer > Lfcin-B (reference peptide) > dimeric. This demonstrated that the dimeric was the least pernicious to human erythrocytes.

**Table 4:** Hemolytic activity of the tested peptides.

**Peptide**	**Alternate name**	^a^**H**_**max**_		**^b^H_50_ (μM)**
		**(%)**	**Peptide Concentration (μM)**	
LfcinB (20–25)	Monomer	7.1	25	>100
PLS	Palindromic	24.8	100	>100
LfcinB (17–31)	Reference Peptide	6.6	25	>100
LfcinB (20–25)_2_	Dimeric	5.6	100	>100
LfcinB (20–25)_4_	Tetrameric	49.1	100	>100

a *H_max_.: Maximum hemolytic activity attained of human red blood cells after 2 h of treatment at 37°C with each peptide molecule*.

*Peptide concentration: concentration (μM) of peptide corresponding to H_ma_*.

b *H_50_: concentration of peptide (μM) leading to 50% hemolysis of human red blood cells after 2 h of treatment at 37°C*.

### Antimicrobial activity on clinical isolates of HCAI pathogens

Having investigated the antibacterial activity of the peptides on diverse bacterial cell lines, we next sought to assess their activity against Gram-positive and Gram-negative bacteria from the 20 HCAI clinical isolates. We tested four species in total: *E. faecium* and *S. aureus* (Gram-positive) and *K. pneumoniae* and *P. aeruginosa* (Gram-negative) (Table 5). We did not test *E. faecalis* here because currently, it is relatively rare among the patient population (Bogotá hospital network). Thus, we replaced it with vancomycin-resistant *E. faecium*, a Gram-positive species frequently encountered in the clinic.

**Table 5:** Antibacterial activity of the RRWQWR-based peptides against the clinical isolates of HCAI-relevant bacteria.

**Gram classification**	**Bacteria**	**Isolates**	**MIC**_**50**_ **(μM)**				**MBC (μM)**				**Resistant phenotype**
			**LfcinB (20–25)**	**PLS**	**LfcinB (20–25)_2_**	**LfcinB (20–25)_4_**	**LfcinB (20–25)**	**PLS**	**LfcinB (20–25)_2_**	**LfcinB (20–25)_4_**	
			**Monomer**	**Palindromic**	**Dimeric**	**Tetrameric**	**Monomer**	**Palindromic**	**Dimeric**	**Tetrameric**	
Gram-positive	*Enterococcus faecium*	550	>100	>100	>100	>100	>100	100.0	>100	12.5	STR, CIP, LVX, ERY, TEC, VAN, TET, SXT
		1,040	>100	>100	>100	>100	>100	>100	>100	>100	STR, CIP, LVX, ERY, TEC, VAN, TET, TMS
		1,225	>100	>100	>100	>100	>100	100.0	>100	12.5	STR, CIP, LVX, ERY, TEC, VAN, TET, TMS
		1,461	>100	>100	>100	>100	>100	100.0	>100	12.5	STR, CIP, LVX, ERY, TEC, VAN, TET, TMS
		1,462	>100	5.7	49.8	>100	>100	100.0	>100	12.5	STR, CIP, LVX, ERY, TEC, VAN, TET y TMS
	*Staphylococcus aureus*	52,013	5.1	8.3	12.6	6.7	>100	100.0	25.0	12.5	Sensitive
		43,062	5.1	50.4	13.5	6.7	>100	100.0	50.0	12.5	OXA
		43,337	3.7	13.0	13.5	6.3	>100	50.0	25.0	12.5	OXA
		48,575	3.7	36.5	13.4	9.2	>100	100.0	25.0	12.5	CLI
		48,577	3.7	5.7	12.9	6.3	>100	100.0	50.0	100.0	OXA
Gram-negative	*Klebsiella pneumoniae*	49,644	>100	12.5	12.3	ND	>100	100.0	50.0	ND	IPM, MEM, ETP, DOR, FEP, CAZ, CRO, TZP, CIP
		50,181	>100	>100	6.6	5.5	>100	>100	100.0	100.0	IPM, MEM, ETP, DOR, FEP, CAZ, CRO, TZP
		50,424	11.3	5.6	5.8	7.8	>100	>100	>100	100.0	IPM, MEM, ETP, DOR, FEP, CAZ, CRO, TZP, GEN, CIP
		51,048	>100	11.7	5.7	5.2	>100	>100	100.0	50.0	IPM, MEM, ETP, DOR, FEP, CAZ, CRO, TZP
		51,009	>100	25.6	5.6	1.6	>100	100.0	25.0	25.0	MEM, ETP, CTX, CRO, AMK, GEN, SXT, FEP, CAZ y CIP, NOR, NIT
	*Pseudomonasss aeruginosa*	47,661	>100	75.0	13.4	7.5	>100	>100	>100	25.0	MEM, IPM, FEP, CAZ, CIP, AMK, GEN, TGC
		48,220	>100	>100	50.0	12.6	>100	>100	50.0	25.0	Sensitive
		48,221	>100	>100	64.2	11.9	>100	>100	>100	50.0	MEM, FEP, CAZ, AMK, CIP
		48,458	>100	>100	75.0	72.8	>100	>100	>100	>100	MEM, GEN, CAZ, FEP, AMK
		48,526	>100	>100	36.3	24.9	>100	100.0	>100	>100	Sensitive

*Amikacin (AMK), cefepime (FEP), cefotaxime (CTX), ceftriaxone (CRO), ceftazidime (CAZ), ciprofloxacin (CIP), clindamycin (CLI), doripenem (DOR), ertapenem (ETP), erythromycin (ERY), gentamicin (GEN), imipenem (IPM), levofloxacin (LVX), meropenem (MEM), oxacillin (OXA), piperacillin-tazobactam (TZP), streptomycin (STR), teicoplanin (TEC), tetracycline (TET), tigecycline (TGC), trimethoprim-sulfamethoxazole (SXT), vancomycin (VAN)*.

*ND: Not determined*.

According to the MIC_50_ and MBC values, the monomer RRWQWR was active primarily against *S. aureus*; the palindromic peptide, predominantly against *S. aureus* and *K. pneumoniae*; and the dimeric and tetrameric peptides had the widest antibacterial spectra and strongest activities, inhibiting *S. aureus, K. pneumonia*, and *P. aeruginosa*. Thus, based on MIC_50_ values, the overall activity ranking for these peptides against all clinical isolates was, from strongest to weakest: tetrameric > dimeric > palindromic > monomer. However, the MBC values give a different picture. Firstly, the monomer was not effective against any of the bacteria. Secondly, the palindromic peptide was active against all four species, as follows (from highest inhibition to lowest): *S. aureus* > *K. pneumoniae* > *E. faecium* = *P. aeruginosa*. The dimeric peptide was active against all the isolates except for one *E. faecium* sample. And, again, the tetrameric peptide was strongly active against all the isolates (from highest inhibition to lowest): *S. aureus* > *K. pneumoniae* > *E. faecium* > *P. aeruginosa*. Interestingly, the tetrameric peptide was highly specific for the Gram-positive isolates.

### Therapeutic index

The therapeutic index (TI) is a ratio of the toxic dose of a substance to its therapeutically-active dose and can be calculated different ways (e.g., LD_50_/ED_50_). Here, we calculated a TI value for each peptide against all the Gram-positive or the Gram-negative ATCC strains, by dividing its H_max_ by its MIC_50_ for the given group of strains. Since the tetrameric peptide was consistently the most active, here we report the TI values for the other peptides relative to its value, using fold values. Additionally, to make our quantitative analysis more robust [geometric mean (Khachatryan et al., 2017) and fold values], we have included MIC_50_ values for these peptides against *S. aureus* and *K. pneumoniae* that we previously obtained using the same assay, the M7-A7 method of the National Committee for Clinical Laboratory Standards (Leon-Calvijo et al., 2015).

Firstly, we calculated separate TI values for each peptide against all the Gram-positive or all the Gram-negative ATCC strains (Table 6). The tetrameric peptide had the highest TI value, suggesting that it may have a wide therapeutic window for antibacterial use, particularly against Gram-positive bacteria.

**Table 6:** Therapeutic Index values for the RRWQWR-based peptides against the ATCC strains of HCAI-relevant bacteria.

**Peptide**	**Attribute**	^**a**^**H**_**max**_		**Gram positive**							**Gram negative**							
			**MIC (**μ**M)**				**^g^Gm**	**^h^Fold**	^**i**^**Therapeutic index**		**MIC (**μ**M)**				**^g^Gm**	**^h^Fold**	^**i**^**Therapeutic index**	
		**(%)**	**(μM)**	**^b^29212-^c^S**	**^d^25923-^c^S**	**^e^33591-^f^R**			**MHC/MIC**	**^j^Fold**	**^k^13883-^c^S**	**^l^700603-^f^R**	**^m^10145-^c^S**	**^n^27853-^f^R**			**MHC/MIC**	**^j^Fold**
LfcinB (20–25)	Monomer	7.1	25.0	>200.0	>200.0	>200.0					>200.0	>200.0	>200.0	>200.0				
PLS	Palindromic	24.8	100.0	25.6	34.9	25.0	28.2	9.0	3.6	0.1	30.7	26.2	99.7	107.2	54.1	4.1	1.8	0.2
LfcinB (17–31)	Lfc B reference peptide	6.6	25.0	34.3	>200.0	50.0	414.4	13.2	0.6	0.0	>200.0	>200.0	111.7	99.6	105.5	8.1	0.2	0.0
LfcinB (20–25)_2_	Dimeric	5.6	100.0	13.1	3.0	24.1	9.8	3.1	10.2	0.3	9.0	7.3	29.1	34.8	16.1	1.2	6.2	0.8
LfcinB (20–25)_4_	Tetrameric	49.1	100.0	3.1	1.7	5.9	3.1	1.0	31.8	1.0	12.0	6.2	18.1	21.7	13.1	1.0	7.6	1.0

a *H_max_: Maximum Hemolytic Activity of the indicated peptide against human erythrocytes after 2 h of treatment at 37°C*.

b *29212: Enterococcus faecalis*.

c *S: sensitive strain*.

d *25923: Staphylococcus aureus*.

e *33591: Staphylococcus aureus*.

f *R: resistant strain*.

g *GM: geometric mean of MIC_50_ values from all three Gram-positive or Gram-negative bacterial ATCC strains in the table*.

h *Fold: Calculated as (GM for the indicated peptide)/(GM for the tetrameric peptide)*.

i *Therapeutic Index: H_max_/GM for the peptides against the Gram-positive or Gram-negative bacteria studied here. A larger value correlates to greater antimicrobial specificity*.

j *Fold: Calculated as (TI for the indicated peptide)/(TI for the tetrameric peptide)*.

k *13883: Klebsiella pneumonia*.

l *700603: Klebsiella pneumoniae*.

m *10145: Pseudomonass aeruginosa*.

n *27853: Pseudomonass aeruginosa*.

Finally, we determined the TI values of the three most active peptides from the previous experiments against four of the clinical isolates (two Gram-positive bacteria and two Gram-negative bacteria). We did not calculate values for the monomer, as it was generally inactive against the ATCC strains and the isolates. The results are shown in Table 7 (Gram-positive) and Table 8 (Gram-negative). Regarding the Gram-positive bacteria, the palindromic, dimeric and tetrameric peptides were active chiefly against *S. aureus*. This trend was consistent with results of the experiments on the ATCC strains, in which these peptides were only active against the sensitive strain of the *Enterococcus* bacteria. The GM values demonstrate that the tetrameric peptide was active at lower doses than were the palindromic or dimeric peptides, which had similar potencies. Calculating the fold-MIC_50_ values relative to the MIC_50_ value for the tetrameric peptide gave values of 2.4 for the dimeric peptide and 2.0 for the palindromic peptide. Taken together, the observed values for GM, MIC_50_, and TIC against the clinical isolates suggest that the tetrameric peptide has the strongest antibacterial activity.

**Table 7:** Therapeutic Index values for the RRWQWR-based peptides against the clinical isolates of HCAI-relevant, Gram- positive bacteria.

**Peptide**	**Attribute**	^**a**^**H**_**max**_		**MIC**_**50**_ **(**μ**M)**										**bGm**	**^c^Fold**	**Therapeutic index**	
		**(%)**	**(μM)**	***Enteroccocus faecium***					***Staphylococcus aureus***							**MHC/MIC_50_**	**^d^Fold**
				**550**	**1040**	**1225**	**1461**	**1462**	**52013**	**43062**	**43337**	**48575**	**48577**				
PLS	Palindromic	24.8	100.0					5.7	8.3	50.4	13	36.5	5.74	13.7	2.0	7.3	0.5
LfcinB (20–25)_2_	Dimeric	5.6	100.0					49.8	12.6	13.5	13.5	13.4	12.9	16.4	2.4	6.1	0.4
LfcinB (20–25)_4_	Tetrameric	49.1	100.0						6.7	6.7	6.3	9.2	6.3	7.0	1.0	14.4	1.0

a *H_max_: Maximum Hemolytic Activity of the indicated peptide against human erythrocytes after 2 h of treatment at 37°C*.

b *GM: geometric mean of the MIC_50_ values for the indicated peptide against the indicated bacterial strains*.

c *Fold: Calculated as (GM for the indicated peptide)/(GM for the tetrameric peptide)*.

d *Fold: Calculated as (TI for the indicated peptide)/(TI for the tetrameric peptide)*.

**Table 8:** Therapeutic Index values for the RRWQWR-based peptides against the clinical isolates of HCAI-relevant, Gram- negative bacteria.

**Peptide**	**Attribute**	^**a**^**H**_**max**_		**MIC**_**50**_ **(**μ**M)**										**^b^Gm**	**^c^Fold**	**Therapeutic index**	
		**(%)**	**(μM)**	***Klebsiella pneumoniae***					***Pseudomonas aeruginosas***							**MHC/MIC_50_**	**^d^Fold**
				**49644**	**50181**	**50424**	**51048**	**51009**	**47661**	**48220**	**48221**	**48458**	**48526**				
PLS	Palindromic	24.8	100.0	12.5		5.6	11.7	25.6	75.0					17.4	1.8	5.8	0.6
LfcinB (20–25)_2_	Dimeric	5.6	100.0	12.3	6.6	5.8	5.7	5.6	13.4	50.0	64.2	75.0	36.3	16.8	1.7	6.0	0.6
LfcinB (20–25)_4_	Tetrameric	49.1	100.0		5.5	7.8	5.2	1.6	7.5	12.6	11.9	72.8	24.9	9.7	1.0	10.4	1.0

a *H_max_: Maximum Hemolytic Activity of the indicated peptide against human erythrocytes after 2 h of treatment at 37°C*.

b *GM: geometric mean of the MIC_50_ values for the indicated peptide against the indicated bacterial strains*.

c *Fold: Calculated as (GM for the indicated peptide)/(GM for the tetrameric peptide)*.

d *Fold: Calculated as (TI for the indicated peptide)/(TI for the tetrameric peptide)*.

Regarding the Gram-negative bacteria, the palindromic, dimeric, and tetrameric peptides were all active *K. pneumoniae* and *P. aeruginosa* (Table 8). As indicated by the GM values, the tetramermic peptide was the most active and the palindromic peptide, the least. Calculating the fold-MIC_50_ relative to the MIC_50_ for the tetrameric peptide gave values of 1.7 for the dimeric peptide and 1.8 for the palindromic peptide. The tetrameric peptide again had the highest TI value, which was even higher than its TI value against Gram-positive bacteria. All together, these values suggest that the tetrameric peptide is the most active of the peptides against Gram-negative bacteria.

## Discussion

The antibacterial activity of AMPs has been correlated to physicochemical properties such as net charge and hydrophobicity. For instance, the cationic segments of AMPs are known to favor electrostatic attraction, thereby driving the peptides toward negatively-charged components on bacterial membrane surface (Shang et al., 2012; Ma et al., 2014; Chen et al., 2015). However, the relationship between charge and antibacterial activity is not linear: above a certain threshold (usually, +6), increasing the positive charge does not improve activity (Dathe et al., 2001; Park and Hahm, 2012; Yin et al., 2012). Given that in our five peptides, net charge was directly proportional to the number of RRWQWR motifs (tetrameric > dimeric > reference > palindromic = monomer), then by extension, higher net charge appeared to correlate to stronger bacterial activity (tetrameric > dimeric > monomer). Indeed, our two most active AMPs, with net charges of +12 (tetrameric) and +6 (dimeric), exhibited strong activity against seven of the eight ATCC bacterial strains (MIC_50_: 1.7–21.7 μM) and against 17 of the 20 clinical isolates (1.6–73.8 μM for clinical isolates).

The hydrophobicity of our peptides might also have influenced their activity. We designed the two branched RRWQWR-based peptides by linking each pair of monomers to a shared Lys residue in the linker, which also included one or two residues of Ahx, a common hydrophobic spacer that prevents steric hindrance (Leon-Calvijo et al., 2015). The short sequence RRWQWR contains an interesting combination of hydrophobic (W, tryptophan) and cationic (R, arginine) amino acids (Table 2). Our results corroborated a direct link between the proportion of hydrophobic residues and the activity. Thus, among the linear peptides, the palindromic peptide (44.4% hydrophobic residues) was more active against the ATCC strains (seven of eight; Table 3) and the clinical isolates (fourteen of 20; Table 5) than was the reference peptide (33.3% hydrophobic residues) or the monomer (33.3% hydrophobic residues).

Finally, from a synthetic perspective, among the three most active peptides (tetrameric > dimeric > palindromic), the two branched peptides were easier to prepare, as they implied fewer coupling steps (9 for the tetrameric and 8 for the dimeric, compared to 9 for the palindromic). This practical advantage, combined with their superior activity, contributes to their attractiveness as starting points for possible antibacterial agents. Our results are consistent with those of previous reports that branched short peptides are more active than linear ones (Lopez-Garcia et al., 2002; Park and Hahm, 2012; Pires et al., 2015).

Although we did not screen the five peptides against many bacterial species, our objective was merely to establish a preliminary assessment of their antibacterial activities against a small variety of antibiotic-sensitive and antibiotic-resistant Gram-positive and Gram-negative bacteria relevant to HAIs.

Among the most surprising results that we observed with the ATCC lines was that at certain concentrations, some of the peptides induced growth of certain strains (Figure 2). This might simply reflect the diverse effects that AMPs and bacteria can have on each other, including proteolytic degradation of peptides by bacterial enzymes (peptidases and proteases) (Schmidtchen et al., 2002), as has been reported by other authors studying LfcinB-derived peptides in *E. faecalis* and other bacteria (Schmidtchen et al., 2001). Thus, such peptides must be studied carefully to determine their proper therapeutic window, which may be rather narrow. This might simply reflect an inherent lack of activity of LfcinB-derived peptides against the entire *Enterococcus* genus. Curiously, in our study, the monomer was inactive against the ATCC strains (Table 3); however, in previous reports, it was shown to be active against the same sensitive strain of *E. faecalis* that we tested (ATCC 29212; MIC_50_: 101.5 μM) (Leon-Calvijo et al., 2015). This discrepancy underscores that, while ATCC lines can be useful tools for assaying antimicrobial activity, they are not definitive indicators of activity, which must be assessed using clinical isolates.

Our SEM analysis revealed that the dimeric and tetrameric peptides induced changes in the sensitive strain of *E. faecalis* (Figure 3) only, and in both the sensitive and resistant strains of *P. aeruginosa* (Figure S1). These results agree with those obtained for other cationic peptides studied at the surface of these bacteria (Winfred et al., 2014; Spitzer et al., 2016), which suggest that the mechanism of action of each peptide involves the membrane. Interestingly, our observations that each peptide induced damage and porosity in the membrane of *P. aeruginosa* (Figure S1), mirror literature reports on other AMPs (Benli and Yigit, 2008; Cao et al., 2017). Also the SEM microphotographys display how *P. aeruginosa* has not surface biofilm As others authors has been demonstrate that Lactoferrin has anti-biofilm activity interfering with its formation and promoting the formation of thin, flat biofilm, allowing *P. aeruginosa* be more susceptible (Chung and Khanum, 2017).

Our results on the clinical isolates confirmed some of the results that we observed with the ATCC reference strains. Among the most important results was that against the clinical isolates of *E. faecium*, the peptides were either inactive or had MIC_50_ values of at least 100 μM (Table 5), similarly to their activity against the ATCC reference strain of antibiotic-sensitive *E. faecalis*. It was interesting to find again that in terms of activity against *Enterococcus*, the palindromic molecule was more active than the dimer (Table 5). This result open new overview because it could indicate that lineal and palindromic repetition of the short motif may useful design as antibacterial molecules for this gender of bacteria. A recent World Health Organization study has underscored the challenge of developing of antibacterials active against *P. aeruginosa* (WHO, 2014). Thus, among our most encouraging findings, was that the dimeric peptide and the tetrameric peptide were each active against *P. aeruginosa*. These results gave further evidence of the therapeutic potential of these two branched peptides and suggest that might exhibit specificity against Gram-positive species.

Considering our all our findings, we propose here that our dimeric and tetrameric have the following mode of action to inhibit bacterial growth: their large net cationic charge enables them to attach to the bacterial membrane surface, where they create small, permeable holes that disrupt the membrane and provoke cell permeation. The superior activity of these branched peptides relative to the three other RRWQWR-based peptides is consistent with previous reports that branched peptides are more active than linear ones (Tam, 1988; Pires et al., 2015), including a study on antigenic peptides derived from human Lfcin (Azuma et al., 1999).

Although the tetrameric peptide was nearly always the most active in all the assays, it also exhibited the highest hemolytic activity (Hmax: 49.1% = 8x that of the dimer). Hemolytic activity is directly related to the net positive charge of the molecule, which for the tetrameric peptide was +12. Interestingly, we attributed the antibacterial activity of this peptide to this very charge. Our first attempt to reduce the hemolytic activity was to synthesize the dimeric peptide, whose net charge (+6) is half that of the tetrameric peptide. Encouragingly, the dimeric peptide exhibited similar antibacterial and lower hemolytic activity relative to the tetrameric peptide. In terms of future work, one strategy to reduce hemolytic effects would be to explore controlled-release systems for the tetrameric, dimeric or other peptide, whereby the concentration of the released peptide could be controlled temporally to maximize therapeutic efficacy while minimizing hemolytic effects. Another option would be to explore the use of prodrugs and/or peptide conjugates, to improve specific targeting. Examples of such prodrugs include a bioactive peptide linked to delivery peptides or cell-penetrating peptides (Mishra et al., 2017).

Intriguingly, during our experiments using Muller Hinton Broth and the tetrameric peptide at concentrations of 100 and 200 μM, the peptide appeared somewhat unstable: upon addition of the peptide solution, the culture developed turbidity, which disappeared with time. This effect may be down to the salt content in Muller Hinton Broth, as various AMPs have been reported to lose activity in physiological salt solutions and in sera (Goldman et al., 1997; Lee et al., 1997; Wu et al., 1999; Rothstein et al., 2001). Further studies salt interactions and serum binding will be required to determine the utility of the tetrameric peptide, whose use as antimicrobial agent may currently be limited to lower concentrations (hemolytic activity at 12.5 μM: 11.2%).

## Conclusion

We have reported the design, synthesis and screening of a set of short, cationic, LfcinB-derived peptides containing at least one RRWQWR motif, as antibacterial agents against ATCC reference strains and clinical isolates of Gram-positive and Gram-negative bacteria associated with HAIs. Our findings suggest that the branched dimeric peptide is the most attractive candidate for further development: although it was generally less active than the branched tetrameric peptide, it was far less hemolytic and did not suffer from the stability problems that the latter peptide showed in culture. We are currently performing detailed membrane, cellular and systemic toxicity studies on both peptides.

## Ethics statement

This study was approved by the Ethics Committees of the Universidad Nacional de Colombia and the Secretaría de Salud de Bogotá. All patient records were anonymized prior to analysis.

## Author contributions

SV and JR contributed conception and design of the study; DM synthesized the peptides molecules; SV performed *in vitro* assays and SEM microscopy of ATCC strains. SV, JR, and HV contributed conception and design of the clinical isolates test. MC and SV performed the *in vitro* assay with clinical isolates; SV wrote the first draft of the manuscript; DM, MC, HV, SV, and JR wrote sections of the manuscript. All authors contributed to manuscript revision, read and approved the submitted version.

### Conflict of interest statement

The authors declare that the research was conducted in the absence of any commercial or financial relationships that could be construed as a potential conflict of interest.

## Supplementary material

Figure S1 Scanning electron microscopy (SEM) images of Gram-positive (*E. faecalis*: Sensitive ATCC-29212; Resistance ATCC-51575) and Gram-negative (*P. aeruginosa:* Sensitive ATCC-10145; Resistance ATCC-27853) strains before and after treatment with the dimeric or tetrameric peptides. **(Top)** The sensitive strain, untreated (left), and after treatment with either the dimeric (middle) or tetrameric (right) peptide at 3 × MIC_50_ for 2h. **(Top)** *E. faecalis*: ATCC-29212 (300.0 and 75.0 μM, dimeric or tetrameric peptides respectively); Resistance ATCC-51575 (200 μM was used because those peptides have not induced MIC_50_ on this strain). **(Bottom)** *P. aeruginosa*: Sensitive ATCC-10145 (87.3 and 54.3 μM, dimeric or tetrameric peptides respectively) and for the Resistance ATCC-27853 (104.4 and 63.3 μM respectively).
