# Supplementary figures and images for: Design, Synthesis and Evaluation of Branched RRWQWR-Based Peptides as Antibacterial Agents Against Clinically Relevant Gram-Positive and Gram-Negative Pathogens
Source: Front Microbiol. 2018 Mar 2;9:329. doi: 10.3389/fmicb.2018.00329 (PMC5840262; doi:10.3389/fmicb.2018.00329)

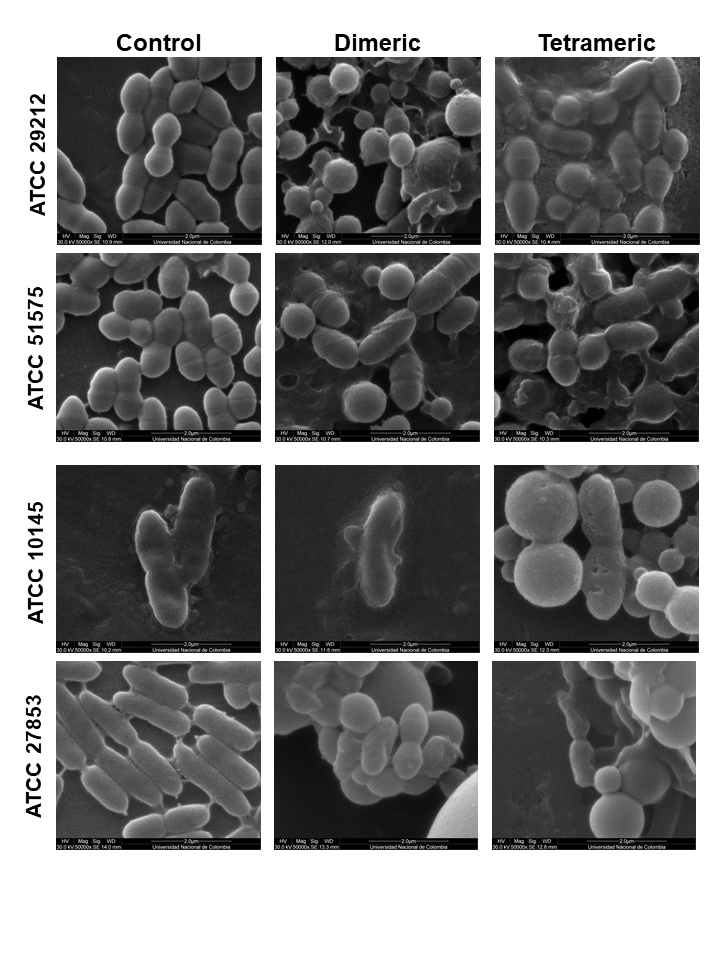

Supplement: Figure S1 — Scanning electron microscopy (SEM) images of Gram-positive (E. faecalis: Sensitive ATCC-29212; Resistance ATCC-51575) and Gram-negative (P. aeruginosa: Sensitive ATCC-10145; Resistance ATCC-27853) strains before and after treatment with the dimeric or tetrameric peptides. (Top) The sensitive strain, untreated (left), and after treatment with either the dimeric (middle) or tetrameric (right) peptide at 3 × MIC50 for 2h. (Top) E. faecalis: ATCC-29212 (300.0 and 75.0 μM, dimeric or tetrameric peptides respectively); Resistance ATCC-51575 (200 μM was used because those peptides have not induced MIC50 on this strain). (Bottom) P. aeruginosa: Sensitive ATCC-10145 (87.3 and 54.3 μM, dimeric or tetrameric peptides respectively) and for the Resistance ATCC-27853 (104.4 and 63.3 μM respectively). [file Image1.TIF]
